# Chemical composition, antimicrobial properties and toxicity evaluation of the essential oil of *Cupressus lusitanica* Mill. leaves from Cameroon

**DOI:** 10.1186/1472-6882-13-130

**Published:** 2013-06-13

**Authors:** Gerald Ngo Teke, Kemadjou Nana Elisée, Kuiate Jules Roger

**Affiliations:** 1 Department of Medicine, Faculty of Health Sciences, University of Bamenda, PO Box 39, Bambili, Cameroon; 2 Department of Biochemistry, Faculty of Science, University of Dschang, PO Box 67, Dschang, Cameroon

**Keywords:** Cupressus lusitanica, Essential oil, Chemical composition, Antimicrobial activity, Toxicity

## Abstract

**Background:**

The leaves of *Cupressus lusitanica* Mill. are used in the western highlands of Cameroon for their medicinal property.

**Methods:**

The leaves of this species were collected in the West Region of Cameroon in August 2010 and subjected to hydrodistillation to obtain the essential oil. The oil was fractionated using adsorption column chromatography. The chemical composition of this oil and its fractions was analysed by gas chromatography–mass spectrometry (GC-MS). The essential oil and fractions were tested for antimicrobial activity against eight bacterial species and six species of *Candida* by the agar diffusion method. Macrodilution method was used to determine the minimum inhibition concentrations (MICs) and minimum bactericidal and/or fungicidal concentrations (MBCs and MFCs). The toxicity profile of the oil was studied using Swiss mice and Wistar albino rats.

**Results:**

Forty-nine compounds were identified in the essential oil. The main components were germacrene D (18.5%), *epi*-zonarene (8.2%), *cis*-calamenene (8.2%), terpinen-4-ol (6.3%), linalool (6.0%) and umbellulone (6.0%). *Enterococcus faecalis*, *Proteus mirabilis* and *Candida albicans* were most susceptible to the oil (MICs of 1.25 and 0.16% for bacteria and fungi respectively). The estimated oral LD_50_ was 6.33 g/kg. There was an increase in sera ALT and AST activities while the blood cells and protein levels decreased in treated animals.

**Conclusion:**

The results obtained from this study support the ethnomedicinal use of *C. lusitanica* leaf oil in the treatment of whooping cough and skin infections though it should be used with care. This plant oil could be useful in the standardisation of phytomedicine.

## Background

Down the ages, essential oils and other extracts of plants have evoked interest as sources of natural products. They have been screened for their potential uses as alternative remedies for the treatment of many infectious diseases [1] since the spread of drug resistant pathogens is becoming a serious threat to successful treatment of microbial diseases. Phytotherapy includes the usage of medicinal and aromatic plants, which constitute a major source of natural organic compounds, to alleviate illnesses. Essential oils have been shown to possess antibacterial, antifungal, antiviral insecticidal and antioxidant properties [2,3] and anticancer activity [4]. There has been an increased interest in looking at antimicrobial properties of extracts from aromatic plants particularly essential oils [5]. Therefore, it is reasonable to expect a variety of plant compounds in these oils with specific as well as general antimicrobial activity and antibiotic potential [6].

These natural compounds are complex and volatile products formed by aromatic plants as secondary metabolites [7]. They are known for their bactericidal, virucidal, fungicidal, sedative, anti-inflammatory, analgesic, spasmolytic, and local anesthetic properties [7]. Chemically, these volatile products and their oxygenated compounds are derived from terpenes [8]. Each of these constituents contributes to the beneficial or adverse effects.

*C. lusitanica* Mill. (Cupressaceae), an essential oil bearing plant, is a tree attaining 25–30 m in height. It was introduced in Cameroon during the colonial era and today is mainly found in the western highlands [9]. The essential oil from the leaves of *C. lusitanica* is commonly used to treat haemorrhoids, rheumatism, whooping cough and styptic problems [10]. The leaves of *C. lusitanica* are used traditionally to protect stored grains from insect infestation and also to cure skin diseases.

It has also been demonstrated that *C. lusitanica* oil has an inhibitory effect against dermatophytes [11]. The essential oil of three samples of *C. lusitanica* from Portugal has been found to be rich in α-pinene, sabinene and abietadiene [12]. In related studies, Floreani et al. [13] reported α-pinene and δ-3-carene as major constituents while Carmo and Frazao [14] revealed that myrcene, sabinene, α-pinene, β-pinene, δ-3-carene were the main constituents. To the best of our knowledge, no scientific work was seen reporting on the toxicity profile of the Cameroonian *Cupressus lusitanica* leaf essential oil.

In the present study, the chemical composition, antimicrobial properties and possible deleterious effects of the essential oil from *C. lusitanica* leaves were investigated.

## Methods

### Plant material and extraction of essential oil

Fresh leaves were collected from non-flowering *Cupressus lusitanica* Mill. plants in the campus of the University of Dschang in August 2010. The plant material was identified at the Cameroon National Herbarium in Yaounde where a voucher specimen is deposited under the code number HNC 66102. The leaves were subjected to hydrodistillation in batches of 2 kg in 3 L of water using a Clevenger-type apparatus. The obtained essential oil was dried over anhydrous sodium sulphate and stored at + 4°C until tested and analyzed.

### Fractionation of essential oil

Five grams of the essential oil was subjected to column chromatography on silica gel (20 g, silica gel 40G, column 30 × 300 mm) with a gradient of hexane-acetyl acetate (95:5, 90:10, 85:15, 80:20, 70:30, 50:50, 25:75), 100% acetyl acetate and methanol. 80 fractions were collected. The band patterns of the fractions were examined on silica gel plates (silice 60G, type F_254_, Alugram® SIL) with hexane-acetyl acetate (8:2 v/v) as the developing reagent. The plates were sprayed with 50% of sulphuric acid and heated at 100°C for band visualization. Fractions showing similar band patterns were combined and a total of seven fractions (F_1_-F_7_) were obtained.

### GC-MS analysis conditions

The GC/MS analysis of the essential oil was performed using a Hewett-Packard 5890II GC, equipped with a HP-5 MS capillary spectrometer (30 m × 0.25 mm i.d., 0.25 μm film thickness) and a mass system with ionization energy of 70 ev. Helium was the carrier gas at a flow rate of 1 ml/min. Injector and MS transfer line temperatures were set at 220 and 290°C, respectively. Column temperature was gradually increased from 50 to 150°C at a rate of 3°C/min, and then held for 10 min. Finally, it was raised to 250°C at 10°C/min. Diluted samples (1:100 v/v in hexane) of 1.0 μl were injected manually in the split-less mode. The components were identified by comparing their relative retention times and mass spectra with those of standards, NBS75K library data of the GC/MS system, and literature data [15]. The results were further confirmed by comparing the elution order of the compounds with their relative retention indices on non-polar phases as reported by Adams [15].

### Microorganisms and growth conditions

The microorganisms used in this study consisted of two Gram (+) bacteria- *Enterococcus faecalis* ATCC 10541 and *Staphylococcus aureus* ATCC 25922; six Gram (−) bacteria- *Escherichia coli* ATCC 11775, *Klebsiella pneumoniae* ATCC13883, *Proteus mirabilis, Pseudomonas aeruginosa* ATCC 27853, *Salmonella typhi* ATCC 6539 and *Shigella flexneri*; and 6 fungi- *Candida albicans* ATCC 9002, *C. glabrata* IP 35, *C. krusei* ATCC 6258, *C. lusitaniae* ATCC 200950, *C. parapsilosis* ATCC 22019 and *C. tropicalis* ATCC 750. The reference strains (ATCC) were obtained from American Type Culture Collection. The two clinical bacterial isolates were collected from Pasteur Center (Yaounde-Cameroon) and the candidal strain was obtained from Pasteur institute (IP, Paris-France). The bacterial and candidal strains were grown at 35°C and maintained on nutrient agar (NA, Conda, Madrid, Spain) and Sabouraud Dextrose Agar (SDA, Conda) slants respectively.

### Antimicrobial assay

The essential oil and its fractions from *C. lusitanica* were tested for antimicrobial activities using agar disc diffusion technique to determine the diameter of growth inhibition zones while broth macrodilution method was used to determine the MIC and MBC/MFC.

### Agar diffusion test

The disc diffusion method [16] was employed for the determination of antimicrobial activities of essential oil and its fractions (F1, F3, F4 and F5). Briefly, 0.1 ml of suspension of the test microorganisms containing 1.5 × 10^7^ cfu/ml of bacteria or 1.5 × 10^6^ spores/ml was spread on Mueller Hinton agar or Sabouraud dextrose agar medium respectively. Filter paper discs (Whattmann n°3, 6 mm in diameter) were soaked with 10 μl of the different concentrations of oil (100%, 10%, 0.1%v/v) and fractions (10%, 1%, 0.1%v/v), and placed on the seeded plates. A negative control was prepared using the solvent (10% DMSO) employed to dissolve the oil. Gentamicin and nystatin (10 μg/disc) were used as positive reference drugs for bacteria and fungi respectively. The fractions F2, F6 and F7 were not tested in this assay due to insufficient quantities. The plates were incubated at 37°C (24 hrs) for bacterial strains, and 35°C (48 hrs) for yeast. Antimicrobial activity was evaluated by measuring the diameters of inhibition zones. Each assay in this experiment was repeated three times.

### Broth macrodilution tests

The minimal inhibitory concentration values were determined for all bacterial and candidal strains using the macrowell dilution method as described by Zgoda and Porter [17]. The inocula of the bacterial and fungal strains were cultured 18 hrs at 37°C in nutrient broth (NB) and 48 hrs at 35°C in Sabouraud dextrose broth (SDB) respectively. Tests strains were suspended in sterile saline to give a final density of 1.5 × 10^6^ cfu/ml of bacteria or 1.5 × 10^5^ spores/ml.

The 24 well plates were prepared by dispensing into each well 500 μl of nutrient broth. A 500 μl of *C. lusitanica* essential oil initially prepared at the concentration of 40% v/v was added into each of the first wells, followed by 2-fold serial dilution to obtain concentration range of 10 to 0.08% v/v. To this was separately added 500 μl of the prepared inoculum. The last well containing 500 μl of nutrient broth/Sabouraud dextrose broth without test substance and 500 μl of the inoculum on each strip was used as the negative control. The final volume in each well was 1 ml. The final concentration of Tween 80 in each medium was 0.5%, which did not affect the growth of the tests microorganisms. Gentamicin and nystatin at concentrations ranging respectively from 250–15 μg/ml, and 16–2 μg/ml were used as positive controls. Culture medium + Tween 80 + test oil was used as the sterility control. The fractions F2, F6 and F7 were not tested in this assay due to insufficient quantities. The plates were covered with a sterile plate sealer and incubated under normal atmospheric pressure at 37°C (24 hrs) for bacterial strains, and 35°C (48 hrs) for yeast. The test samples were screened three times against each microorganism. The bacterial or fungal growth was indicated by the presence of turbidity and/or a white “pellet” at the bottom of the well. The minimum inhibitory concentration was determined as the lowest concentration of the test compound that demonstrated no visible growth. The minimum bactericidal/fungicidal concentration (MBC/MFC) was determined by making subcultures of 10 μl of each well showing no turbidity, and negative control on nutrient agar or sabouraud dextrose agar. Subcultures of bacteria and fungi were incubated at 37°C (24 hrs) and 35°C (48 hrs) respectively. The MBC or MFC was considered as the lowest concentration of oil resulting in no growth (death) of the subcultured inoculum compared to the negative control [18].

### Animals used

Male Wistar albino rats (110–120 g) and Swiss mice (22–25 g) were used for the toxicity assay. They were housed in standard plastic cages and provided with food and water *ad libitum.* The studies were conducted according to the ethical guidelines of the Committee for Control and Supervision of Experiments on Animals (Registration no. 173/CPCSEA, dated 28 January, 2000), Government of India, on the use of animals for scientific research.

### Acute toxicity assay

This study was carried out in mice (males) as described by Emerson et al. [19]. Swiss albino mice of age 7–8 weeks were chosen at random and assigned to 5 different groups of 6 mice each (3 per cage). Food and water were withdrawn 18 hrs before the oral administration of 0.8 ml oil solution to the mice at the doses 2 to 8 g/kg of bodyweight (with a constant increment of 2). The fractions were not tested in this assay due to insufficient quantities. The control animals received 0.8 ml solution of 8% DMSO. The animals were observed for mortality and behavioural responses for 48 hrs, after which the number of deaths in each group was noted. The effect of the oil was assessed on the basis of mortality, expressed as median lethal dose (LD_50_). The LD_50_ value of the essential oil solution was evaluated by calculation [20].

### Subacute toxicity assay

For this assay, 30 Wistar rats (males) aged 8 weeks were used. They were chosen at random and assigned to 5 different groups of 6 rats each (2 per cage). The control group received 0.5 ml of 8% DMSO solution while the test groups received corresponding doses of essential oil solution (from 0.8 to 2 g/kg with an increment of 0.4). All the animals were orally gavaged daily for four weeks and were observed for physiological, behavioural responses and for mortality.

Twenty-four hours after the last administration, the rats were anaesthetized and blood collected by cardiac puncture into heparinised and non-heparinised centrifuge tubes to evaluate some biochemical parameters and estimate blood cells. The heparinised blood was used to verify total red blood cell and white blood cell counts [21], and heamatocrit level [22]. Serum was prepared from non-heparinised blood and assessed for alanine aminotransferase (ALT), asparatate aminotransferase (AST), alkaline phosphatase (ALP) and creatinine levels using the commercial kits (BioSystems reagents and instruments). Protein levels in sera and organ homogenates were evaluated by the Biuret’s method [23].

### Statistical analysis

The data were subjected to one-way analysis of variance, and differences between samples at *P ≤ 0.05* were determined by Student-Newmann-Keuls multiple range test using the Statistical Package for the Social Sciences (SPSS) program. Experimental results were expressed (where appropriate) as mean ± SEM (standard error to the mean).

## Results

### Chemical composition of essential oil and its fractions

GC/MS analysis of the crude oil (yield of 0.32%) resulted in the identification of 49 compounds representing 83.3%. Germacrene D (18.5%) *epi*-zonarene (8.2%), *cis*-calamenene (8.2%), terpinen-4-ol (6.3%), linalool (6.0%) and umbellulone (6.0%) were the main components, comprising 53.2% of the oil (Table 1). Fractions F1 and F3 were rich in composition than the other fractions.

**Table 1:** **Chemical compounds (%) and retention indices (RI) of *C. lusitanica *Mill. leaf oil and its fractions**

	**Compounds**	**RI**^**a**^		**%**						
			**EO**^**b**^	**F**_**1**_	**F**_**2**_	**F**_**3**_	**F**_**4**_	**F**_**5**_	**F**_**6**_	**F**_**7**_
1	tricyclene	923	0.3	-	-	-	-	-	-	-
2	α-pinene	936	0.6	0.3	-	5.4	-	-	0.6	2.5
3	sabinene	973	0.3	-	-	0.5	-	-	-	-
4	myrcene	987	0.4	0.3	-	-	-	-	-	-
5	δ-3-carene	1001	0.5	0.7	-	1.5	0.3	-	-	-
6	α-phellandrene	1003	0.1	-	-	-	-	-	-	-
7	α-terpinene	1014	0.2	-	-	5.4	-	-	-	-
8	*p*-cymene	1017	0.5	0.9	5.5	1.9	-	-	-	-
9	limonene	1021	2.3	1.6	-	1.0	-	-	1.9	1.4
10	1,8-cineole	1022	0.8	-	-	0.5	0.1	-	-	-
11	γ-terpinene	1057	0.4	-	-	6.8	0.3	-	5.1	-
12	*cis*-linalooloxide	1067	0.5	-	-	-	-	1.2	-	-
13	α -terpinolene	1083	-	0.3	-	-	-	-	-	-
14	linalool	1095	6.0	-	-	0.5	6.4	-	-	-
15	umbellulone	1164	6.0	-	-	2.8	5.9	-	3.6	-
16	terpinen-4-ol	1171	6.3	-	-	1.6	26.3	-	2.7	-
17	*p*-cymen-8-ol	1178	1.0	0.2	-	-	-	-	2.1	1.4
18	*p*-menth-2-en-1-ol	1213	0.3	-	-	-	10.5	-	-	-
19	citronellol	1229	0.2	-	-	-	-	-	-	-
20	linalyl acétate	1250	1.2	-	-	11.3	-	-	4.3	-
21	thymol	1254	0.4	-	-	2.7	0.6	-	-	-
22	α -cubebene	1346	0.3	-	-	10.0	-	4.7	3.1	-
23	α-copaene	1373	0.1	0.5	-	3.5	-	-	-	-
24	α-cedrene	1403	0.1	0.3	-	-	9.5	-	-	-
25	β-caryophyllene	1411	1.5	-	-	-	-	-	-	-
26	β-cedrene	1413	0.4	9.1	-	-	-	-	-	-
27	α-humulene	1448	0.6	0.9	-	-	-	-	-	-
28	zingiberene	1472	0.1	0.3	-	-	-	-	-	-
29	α-curcumene	1478	4.1	11.6	5.3	22.6	-	-	3.6	7.3
30	germacrene D	1481	18.5	-	-	-	1.9	-	-	13.9
31	α-amorphene	1482	2.0	6.9	-	-	-	-	-	-
32	*epi*-zonarene	1498	8.2	-	-	-	-	4.7	3.1	-
33	α-muurolene	1501	1.1	1.9	-	-	-	-	-	-
34	cuparene	1503	-	1.7	-	-	-	-	-	-
35	β-bisabolene	1504	0.3	1.8	-	-	-	-	-	-
36	α-cadinene	1510	0.2	-	-	-	0.6	-	-	-
37	γ-cadinene	1511	-	-	-	-	2.0	-	-	-
38	*cis*-calamenene	1521	8.2	20.1	-	-	-	-	-	-
39	α-calacorene	1530	0.5	0.4	-	-	-	-	-	-
40	spathulenol	1576	0.2	-	2.8	-	-	-	-	-
41	caryophyllene oxide	1584	0.6	0.6	-	10.4	-	-	3.6	-
42	cedrol	1591	1.2	-	-	-	17.2	7.6	-	-
43	di-*epi*-α-cedrene	1633	4.9	1.2	-	-	-	14.8	-	-
44	14-norcadin-5-en-4-one	1696	0.9	4.5	32.0	-	-	-	6.3	4.3
45	*trans*-nuciferol	1718	-	-	4.9	-	-	-	-	-
46	naphthalene	1780	0.8	0.4	17.6	-	4.3	3.2	3.6	5.2
47	kaur-15-ene	1974	0.2	0.4	-	-	-	-	-	-
48	*cis*-totarol	2277	-	-	-	1.6	0.2	-	-	-
49	ferruginol	2324	-	-	-	0.5	-	-	-	-
Monoterpene hydrocarbons			5.6	4.1	5.5	22.5	0.6	-	7.6	3.9
Oxygenated monoterpenes			22.7	0.2	-	19.4	49.8	1.2	12.7	1.4
Sesquiterpene hydrocarbons			51.9	57.1	22.9	36.1	18.3	27.4	13.4	26.4
Oxygenated sesquiterpenes			2.9	5.1	39.7	10.4	17.2	7.6	9.9	4.3
*Others*			0.2	0.4	-	2.1	0.2	-	-	-
Total identified			83.3	66.9	68.1	90.5	86.1	36.2	43.6	36.0

^a^ Compounds are listed in order of their elution from an HP-5 column using the homologous series of n-alkanes; ^b^Essential oil (EO), fractions F1 to F7; - Not detected.

### Antimicrobial activities

The crude essential oil of *C. lusitanica* showed antimicrobial activities against all the bacteria and fungi tested. The highest activity was observed on bacteria. The diameters of inhibition zones for bacterial and fungal strains were in the ranges of 11–18 mm and 7–14 mm respectively (Table 2). This activity was highest at 10% v/v of oil and was observed to decrease with concentration. The fractions of this oil were less active and selective compared to the crude oil. These fractions inhibited most the growth of bacteria with diameter of inhibition zones varying from 7–10 mm.

**Table 2:** **Mean diameters**^**a **^**of microbial growth inhibition zones (mm) of test samples**^**b**^

**Microoganisms**	**Samples**															
	**EO**			**F1**			**F3**			**F4**			**F5**			**Reference**^**c**^
**Gram (−) bacteria**	**100%**	**10%**	**1%**	**10%**	**1%**	**0.1%**	**10%**	**1%**	**0.1%**	**10%**	**1%**	**0.1%**	**10%**	**1%**	**0.1%**	**10 μg**
*Escherichia coli* ATCC 11775	12	9	6	6	6	6	9	8	6	8	8	7	9	7	7	21
*Klebsiella pneumoniae* ATCC 13883	11	8	6	8	7	6	8	6	6	9	8	7	8	6	6	20
*Proteus mirabilis*	11	9	6	13	12	11	8	8	6	12	11	10	12	10	9	18
*Pseudomonas aeruginosa* ATCC 27853	13	9	6	9	7	6	9	7	6	7	6	6	7	6	6	24
*Salmonella typhi* ATCC 6539	11	9	6	8	8	6	8	7	6	7	7	6	6	6	6	24
*Shigella flexneri*	18	13	8	12	8	6	10	8	6	9	7	6	8	7	6	23
**Gram (+) bacteria**																
*Enterococcus faecalis* ATCC 10541	16	12	6	12	10	9	10	8	6	10	8	8	13	11	10	20
*Staphylococcus aureus* ATCC 25922	16	11	7	10	8	6	11	8	6	9	7	6	8	7	6	25
**Fungi**																
*Candida albicans* ATCC 9002	13	7	6	6	6	6	6	6	6	6	6	6	6	6	6	22
*Candida glabrata* CIPA 35	6	6	6	6	6	6	6	6	6	6	6	6	6	6	6	23
*Candida krusei* ATCC 6258	10	6	6	6	6	6	6	6	6	6	6	6	6	6	6	18
*Candida lusitaniae* ATCC 200950	13	10	6	12	9	9	11	9	7	12	10	6	14	12	10	33
*Candida parapsilosis* ATCC 22019	7	6	6	6	6	6	6	6	6	6	6	6	6	6	6	24
*Candida tropicalis* ATCC 750	14	7	6	6	6	6	6	6	6	6	6	6	7	6	6	23

The results are the mean values of triplicate tests measured after 24 hrs (at 37°C for bacteria) and 48 hrs (at 35°C for fungi) incubation; ^a^Diameter value of 6 mm is equal to disc diameter, implying no activity; ^b^Essential oil (EO), fractions of essential oil (F1, F3, F4 and F5); ^c^Reference compounds were gentamicin and nystatine for bacteria and fungi respectively.

The MICs and MBCs/MFCs for the crude oil (quantity of fractions was not enough for the assay) are given in Table 3. Generally, all the microorganisms were sensitive to the test sample. The MIC values of oil for bacteria ranged from 1.25- 10% v/v, with *E. faecalis* and *P. mirabilis* being the most sensitive while *P. aeruginosa* was the least sensitive. It was observed that the fungal strains were more susceptible to the leave essential oil (MIC range from 0.16-1.25% v/v) than bacteria. *C. albicans* was the most sensitive fungal strain.

**Table 3:** Minimal inhibitory concentration (MIC)/Minimum bactericidal or fungicidal concentration (MBC or MFC) of test substances

**Microoganisms**	**MIC**		**MBC/MFC**	
**Gram (−) bacteria**	**Essential oil (%)**	**Reference (μg/ml)**	**Essential oil (%)**	**Reference**^**a **^**(μg/ml)**
*Escherichia coli* ATCC 11775	2.5	31.25	2.5	31.25
*Klebsiella pneumoniae* ATCC 13883	5.00	31.25	>10.00	31.25
*Proteus mirabilis*	1.25	125	1.25	250
*Pseudomonas aeruginosa* ATCC 27853	10.00	31.25	>10.00	31.25
*Salmonella typhi* ATCC 6539	2.5	62.50	2.5	125
*Shigella flexneri*	2.5	62.50	2.5	125
**Gram (+) bacteria**				
*Enterococcus faecalis* ATCC 10541	1.25	15.62	2.5	15.62
*Staphylococcus aureus* ATCC 25922	1.25	62.50	2.5	62.50
**Fungi**				
*Candida albicans* ATCC 9002	0.16	2	0.16	2
*Candida glabrata* CIPA 35	1.25	16	1.25	16
*Candida krusei* ATCC 6258	1.25	4	1.25	4
*Candida lusitaniae* ATCC 200950	0.62	2	0.62	>2
*Candida parapsilosis* ATCC 22019	1.25	16	1.25	16
*Candida tropicalis* ATCC 750	1.25	8	1.25	8

The results are the mean values of triplicate tests measured after 24 hrs (at 37°C for bacteria) and 48 hrs (at 35°C for fungi) incubation; ^*a*^ Reference compounds were gentamicin and nystatine for bacteria and fungi respectively.

### Effects of single oral dose of essential oil on mice

The acute toxicity study of *C. lusitanica* leaf oil on mice showed a slight tolerance to the oil solution. For doses ≥ 4 g/kg the animals responded in uncontrolled movements; their respiratory rhythm increased progressively with increase in dose. The oil provoked deaths of mice; 2 g/kg (0/6), 4 g/kg (1/6), 6 g/kg (2/6) and 8 g/kg (6/6), within 48 hours observation period. The lethal dose-50 (LD_50_) of this extract was evaluated as 6.33 g/kg bodyweight.

### Subacute toxicity

After subjecting the rats to a daily treatment of *C. lusitanica* leaf oil for four weeks, the animals expressed some changes in their behavioural profile in the test groups. Decreases in movement, aggressiveness, response to touch and noise, and respiratory rhythm were observed. No death was recorded during this treatment either in the control or in the drug-treated groups.

From haematological and biochemical observations (Table 4), the red blood cells and white blood cells decreased with increase in dose of oil. This decrease was significant at 1.5 and 2 g/kg with respect to the control. In a like manner, the heamatocrit levels also decreased with the rising doses of oil, and were significantly higher in the control animals (45.78 ± 1.61%) with respect to test doses (Table 4). The least level (35.96 ± 1.05%) of heamatocrit was recorded at 2 g/kg dose of oil.

**Table 4:** **Effects of essential oil of *C. lusitanica *on some biochemical parameters in rats**

		**Biochemical parameters**						
**Treatment**	**Dose (g/kg)**	**Red blood cells (×10**^**3**^**/mm**^**3**^**)**	**White blood cells (/mm**^**3**^**)**	**Hematocrit (%)**	**ALT**^**a **^**(U/l)**	**AST (U/l)**	**ALP (U/l)**	**Creatinine (mg/l)**
**Control**	0	8052 ± 451	6456 ±1690	45.78 ±1.61	10.11 ± 2.32	6.63 ± 0.34	14.12 ± 2.17	24.75 ± 1.68
**Essential oil of *C. lusitanica***	0.8	7066 ± 326	6700 ± 689	43.33 ± 1.83	13.46 ± 3.45	9.59 ± 3.10	14.19 ± 0.99	17.50 ± 2.31^*^
	1.2	6908 ± 265^*^	5657 ± 427	42.14 ±1.39*	15.70 ± 3.17^*^	16.65 ± 2.27^*^	12.06 ± 4.52	14.40 ± 3.20^*^
	1.6	6482 ± 279^*^	5640 ± 203	38.73 ±1.17^*^	17.44 ± 3.61^*^	30.00 ± 3.59^*^	10.86 ± 0.00^*^	11.40 ± 3.20^*^
	2	6018 ± 567^*^	3133 ± 689^*^	35.96 ± 1.05^*^	19.79 ± 3.14^*^	32.65 ± 3.61^*^	11.76 ± 0.99^*^	11.00 ± 3.17^*^

Values are means ± SE of six animals. For the same column, values affected by * are significantly different form the control (test of Student-Newmann-Keuls at p > 0.05); ^a^alanine aminotransferase (ALT), asparatate aminotransferase (AST), Alkaline phosphatase (ALP).

In the study of the effects of *C. lusitanica* leaf oil on some serum enzymes, it was observed that the oil provoked a significant increase in transaminase (AST and ALT) levels in serum at doses of 1.2, 1.6 and 2 g/kg. Compared to the control values of AST (6.63 ± 0.34 U/l) and ALT (10.11 ± 2.32 U/l), the dose of 2 g/kg of oil provoked the greatest increase of 32.65 ± 3.61 U/l and 19.79 ± 3.14 U/l levels of the respective enzymes.

The activity of alkaline phosphatase (ALP) in the serum was not significantly different (*P ≤ 0.05*) from the control after the oil treatment at the tested doses (Table 4). The creatinine level in serum decreased progressively with increase in essential oil dose (Table 4). The least value (11.00 ± 3.17 mg/l) observed at 2 g/kg was significantly different from the control (24.75 ± 1.68 mg/l).

The protein levels in the studied organs and serum decreased as the oil dose increased. This decrease was significant for the liver, heart, lungs, kidneys, spleen and serum at the dose 2 g/kg compared to the control (Table 5). The heart (71.66 ± 11.43 mg/g) and kidneys (117.20 ± 5.24 mg/g) were most affected since at 0.8 g/kg (least test dose) these values were significantly lower than the control (81.96 ± 17.32 mg/g and 156.37 ± 4.65 mg/g respectively).

**Table 5:** **Effects of essential oil of *C. lusitanica *treatment on tissue protein levels in rats**

		**Tissues**					
**Treatment**	**Dose**	**Liver**	**Heart**	**Lungs**	**Kidneys**	**Spleen**	**Serum**
	**(g/kg)**	**(mg/g)**	**(mg/g)**	**(mg/g)**	**(mg/g)**	**(mg/g)**	**(g/l)**
**Control**	0	81.96 ± 17.32	119.83 ± 6.97	168.91 ± 5.62	156.37 ± 4.65	123.93 ± 13.73	21.16 ± 0.73
**Essential oil of *C. lusitanica***	0.8	71.66 ± 11.43	110.73 ± 11.56	153.86 ± 5.28	117.20 ± 5.24^*^	123.61 ± 18.41	18.73 ± 1.75
	1.2	63.13 ± 5.55^*^	102.17 ± 9.92	123.03 ± 3.35^*^	112.95 ± 3.78^*^	110.93 ± 12.16	17.58 ± 0.58
	1.6	58.98 ± 4.38*	104.27 ± 5.79	102.01 ± 4.98^*^	82.71 ± 4.26^*^	116.42 ± 20.14	10.24 ± 1.13*
	2	52.65 ± 9.57*	79.43 ± 4.73^*^	100.01 ± 4.25^*^	31.10 ± 6.16^*^	61.43 ± 11.67^*^	9.24 ± 0.50^*^

Values are means ± SE of six animals. For the same column, values affected by * are significantly different form the control (test of Student-Newmann-Keuls at p > 0.05).

## Discussion

The traditional use of plants as medicines provide the basis for indicating which essential oils and plant oils may be useful for specific medical conditions. Historically, many plant oils and extracts, have been used as topical antiseptics, or have been reported to have antimicrobial properties [24]. It is important to investigate scientifically those plants which have been used in traditional medicines as potential sources of novel antimicrobial compounds. Also, the resurgence of interest in natural therapies and increasing consumer demand for effective, safe and natural products means that quantitative data on plant oils and extracts are required. This study was aimed to characterize chemically and evaluate the antimicrobial activity and toxicity profile of *C. lusitanica* leaf essential oil.

The results on chemical characterization of the essential oils from *C. lusitanica* obtained by GC-MS analyses revealed the predominance of germacrene D, *epi*-zonarene, *cis*-calamenene, terpinen-4-ol, linalool and umbellulone. This finding is slightly different with the previous reports of Adams et al. [12] and Kuiate et al. [11]. The former authors noted that α-pinene, sabinene and umbellulone were the major components in the essential oil from the leaves of *C. lusitanica* samples from Portugal, while the latter remarked the presence of α-pinene, umbellulone, germacrene D and *epi*-zonarene as the main components. This difference in composition between the three samples may be affected by the influence of climatic, seasonal factors and harvest areas [25].

The results of the antimicrobial activities showed that, the essential oil of *C. lusitanica* leaves had moderate antimicrobial activities against all the microorganisms (8 bacteria and 6 *Candida*). This oil showed a greater antibacterial activity compared to antifungal activity with the standard method of disc diffusion. Contrary, candidal species were more sensitive in the macrodilution method. The higher activity of essential oil with the macrodilution compared to disc diffusion method may be due to growth conditions [26]. The antimicrobial nature of the essential oil may be attributed to the presence of β-myrcene, limonene, phellandrene, γ-terpinene, caryophyllene and thymol among the constituents [27-29]. The essential oils containing terpenes are also reported to possess antimicrobial activity [30], which are consistent with our present study. In addition, the components in lower amounts may have also contributed to the antimicrobial activity of the essential oils, involving probably some type of synergism with other active compounds [31]. This could explain why the antibacterial activities of the fractions decreased with respect to the crude essential oil. These differences in antimicrobial activities could be linked to their differential chemical compositions. In the present study, the essential oil from *C. lusitanica* leaves was found to be equally effective against both gram-positive and gram-negative organisms though the Gram-positive bacteria were more resistant to the essential oils than gram-negative bacteria [32]. The differences between MIC and MBC or MFC values were not more than 2-fold, suggesting that the activity of essential oil of this plant could be bactericidal and fungicidal [33]. It is worth mentioning that the antimicrobial activity of the essential oil from *C. lusitanica* leaves from Cameroon is herein reported for the first time.

An important characteristic of essential oils and their components is their hydrophobicity, which enable them to partition the lipids of the bacterial cell membrane and mitochondria, disturbing the cell structures and rendering them more permeable [34,35]. Extensive leakage from bacterial cells or the exit of critical molecules and ions will lead to death [36].

The antimicrobial activity of this plant prompted us to evaluate to what extent the oil could provoke deleterious effects. With a lethal dose-50 (LD_50_) of 6.33 g/kg, the essential oil of *C. lusitanica* leaves can be considered less toxic [37]. The decrease in movement, aggressiveness, sensitivity to touch and noise may result from the action of this oil on the central nervous system [38,39]. It was suggested that the death of mice may be related to respiratory failure due to poor ventilation of the lungs. This idea was put forward due to the fact that the breathing rhythm of some of these animals was abnormal as it increased progressively with time after the administration of essential oil. Some of the mice were observed responding as if they were suffocating and/or having difficulty in breathing. This is in corroboration to the findings of Joseph et al. [40].

Transaminases (ALT and AST) are concerned with amino acids metabolism. Large amount of AST are present in the liver, kidneys, cardiac and skeletal muscles, while ALT is found principally in the liver [41]. Small amount of AST are present in the brain, pancreas, and lungs. The serum or plasma levels of both AST and ALT rise whenever there is liver cell damage. The higher activities of both enzymes reflect the greater degree of liver damage [42,43]. An increased serum activity of these enzymes in the present study indicates that the extract may have significant cytotoxic effects on the liver. The extract could affect the permeability of the cell membrane causing the membrane to become leaky. This would then induce the release of these enzymes from the cells into the blood stream, thereby causing the subsequent plasma or serum elevation of the enzymes [44].

The reduction of blood cells observed in the treated animals could be ascribed to oil toxicity linked to bone marrow failure [45] or liver and spleen affection [21]. Rats that received the oil at high doses exhibited decreases in serum total proteins concentration and total protein concentration of the liver on the other studied organs, suggesting tissue injury [19]. The drop in organ and blood protein levels could cause metabolic and physiological variations, susceptible to provoke disequilibria and behavioural problems observed in the rats. The study of possible toxic effects of *C. lusitanica* leaf essential oil is herein being reported for the first time.

## Conclusion

The results of this work show that the *C. lusitanica* essential oil, rich in aromatic compounds, possesses antimicrobial properties, which can be used as a natural antimicrobial agent for human infectious diseases. This oil may exert deleterious effects at high doses. Further studies are necessary to elucidate the mechanism of action of this oil.

## Competing interests

The authors declare that they have no competing interests.

## Authors’ contributions

GN and KE have carried out the experimental part such as selection of essential oils and its chemical characterization, inoculum preparation and antimicrobial evaluation, and toxicity evaluation. KJ supervised the work, evaluated the results and corrected the manuscript for publication. All authors read and approved the final manuscript.

## Pre-publication history

The pre-publication history for this paper can be accessed here:

http://www.biomedcentral.com/1472-6882/13/130/prepub
